# Optimized Lateral Flow Immunoassay Reader for the Detection of Infectious Diseases in Developing Countries

**DOI:** 10.3390/s17112673

**Published:** 2017-11-20

**Authors:** Evdokia Pilavaki, Andreas Demosthenous

**Affiliations:** Department of Electronic and Electrical Engineering, University College London, Torrington Place, WC1E 7JE London, UK; a.demosthenous@ucl.ac.uk

**Keywords:** infectious diseases, lateral flow immunoassay, point of care, ray trace simulation, reader

## Abstract

Detection and control of infectious diseases is a major problem, especially in developing countries. Lateral flow immunoassays can be used with great success for the detection of infectious diseases. However, for the quantification of their results an electronic reader is required. This paper presents an optimized handheld electronic reader for developing countries. It features a potentially low-cost, low-power, battery-operated device with no added optical accessories. The operation of this proof of concept device is based on measuring the reflected light from the lateral flow immunoassay and translating it into the concentration of the specific analyte of interest. Characterization of the surface of the lateral flow immunoassay has been performed in order to accurately model its response to the incident light. Ray trace simulations have been performed to optimize the system and achieve maximum sensitivity by placing all the components in optimum positions. A microcontroller enables all the signal processing to be performed on the device and a Bluetooth module allows transmission of the results wirelessly to a mobile phone app. Its performance has been validated using lateral flow immunoassays with influenza A nucleoprotein in the concentration range of 0.5 ng/mL to 200 ng/mL.

## 1. Introduction

Low and middle income countries usually struggle to provide sufficient healthcare services access to their population [1]. The high cost of developing and maintaining medical centers and the requirement for trained medical personnel to operate in these facilities are some of the reasons that contribute to this problem. As a result, infectious diseases usually thrive in these environments, while the transmission from person to person of the infection can have fatal results for whole families, or even communities. Therefore, the development of point-of-care (POC) devices that are portable, robust, easy to operate, battery powered, and able to monitor and detect infectious diseases is crucial. 

Lateral flow assays (LFAs) have gained increased interest in diagnostic applications due to their numerous advantages that fulfil the World Health Organization (WHO) guidelines for diagnostic tests in developing countries: affordable, sensitive, specific, user-friendly, rapid, robust, equipment free, and deliverable [2,3]. Interfacing these assays with POC readout devices can give quantitative results of the infection concentration in the samples. Limitations in the sensitivity of the LFIAs can be overcome by various methods. Among the most prominent are the optimization of the conjugate conditions and size of the strip [4,5] or the control of the fluid movement on the strip [6].

**Figure 1:**
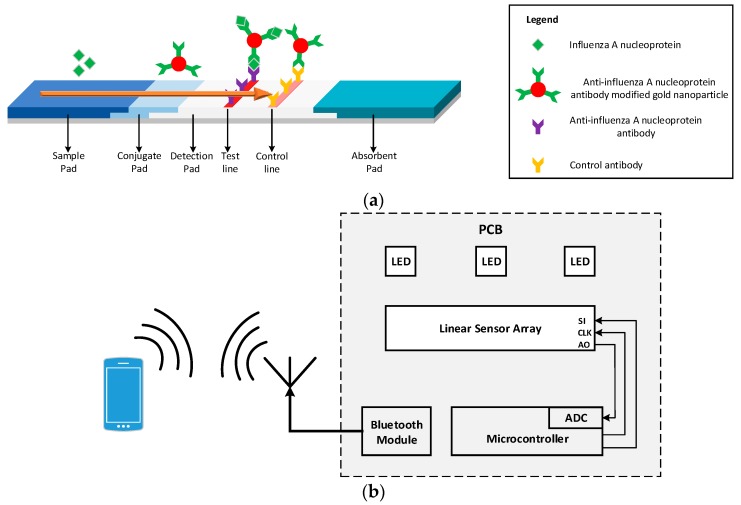
(**a**) Schematic of the LFIA for the detection of Influenza A nucleoprotein; (**b**) Schematic diagram of the reader.

The LFA consists of different overlapping pads that are preprocessed and usually have a strip shape. When antibodies are used as the identification element, the LFA is called lateral flow immunoassay (LFIA) [7]. Figure 1 shows the LFIA in various stages of its operation. The fluid sample initially is placed at the end of the strip, called the sample pad, and due to capillary forces it flows along the entire length of the strip. Once the sample reaches the conjugate pad, the analyte in the sample binds with the labels (colloidal gold nanoparticles conjugated with antibodies) present in this part of the strip generating an immune complex. The immune complex then flows through the detection pad. This pad is a porous membrane where specific antibodies for the detection of analyte have been immobilized, in the form of lines. If the analyte is present in the immune complex, it binds to the antibodies of the detection pad producing a sandwich format and generating a color (test) line [8]. The color intensity relates to the amount of analyte existing in the sample [9]. The rest of the sample continues flowing and binds with the antibodies in the control line, generating another color (control) line the intensity of which can be used for calibration. The control line must be generated regardless of the test line results to validate the proper operation of the assay [5,10]. The excess sample is absorbed in the absorbent pad [9,10]. The results of this process are usually quantitative or semi-quantitative and are interpreted using the colorimetric method [11]. This can be done by either visual inspection of the LFIA or by using a readout device. Figure 1a shows the LFIA for the detection of Influenza A nucleoprotein. 

Many readout devices for the quantification of analyte present in the sample in LFIAs using various methods have been reported. The most common methods are either based on image analysis or on systems that use available discrete components. In the first case, an image of the LFIA is taken—usually using the camera of a mobile phone—and the image is processed using the mobile phone’s resources. Even though these types of readers have fast implementation and are very cheap, they are extremely susceptible to ambient light, focal distance, and tilt [2,8]. An app that uses augmented reality has been introduced in [12] in order to overcome these problems. However, the drawback of this approach is that it does not yield quantitative results. Therefore, in order to tackle these problems and generate quantitative results many use a custom made enclosure around the LFIA and the camera of the mobile phone [13,14,15]. However, in these cases, the systems usually become more complicated and additional optical components are required, like lenses and LEDs. With all the extra optical components that need to be added the main advantage of fast implementation is lost. Also, these systems have reproducibility issues, because different models of mobile phones have different cameras with different resolutions and different lenses which can give different results. A solution to this problem is intensive imaging processing analysis which can result in excess power consumption [16]. 

The other major type of readout device is based only on the use of discrete optical components (lenses, fibers, optical splitters) to direct the light into a photodetector. The results can then be read from a specific part in the strip [17], usually the test line. Alternatively, a mechanical motor can be used to scan a large area in the test strip [18]. However, in the first case (where the readings are taken over a small area) the systems are susceptible to positioning error even at the slightest displacement of the strip [10]. The systems in the second case are usually bulkier, heavier, and fragile, especially when their primary use is to be carried out at the POC for rapid testing. 

This paper presents a handheld optical reader for LFIAs that avoids the drawbacks of these systems. The principle of operation of the system developed is based on shining uniform light in the detection pad surface of the LFIA and measuring the intensity of the reflected light from the LFIA to an array of photodiodes, Figure 1b shows the block diagram of the proposed system. Using this scanning method without the need of any moving parts, the system can quantify the analyte present in the sample for a variety of LFIAs test and control line widths and positions. The device is not affected by ambient light, focal plane, or tilt and the results are reproducible without the need of intensive hardware processing. It is a compact, robust design that uses no moving parts or optical accessories and is ideal for developing countries. The readout device is an improved and optimized system of the preliminary implementation in [8]. Characterization of the surface of a LFIA has been performed in order to investigate the response of light when it impinges the surface of the LFIA. Ray trace simulations have been performed in order to optimize the system in terms of sensitivity and power consumption. The modeled system has been implemented using discrete components; it has been tested using LFIA with Influenza A nucleoprotein in various concentrations. The results are wirelessly transmitted to a mobile phone using Bluetooth. For the calibration of the system, the four parameter logistic model (4PL) [18,19] has been used. The rest of the paper is organized as follows. Section 2 presents the modeling and optimization of the device, while Section 3 describes its physical implementation. Measured results are presented in Section 4 followed by concluding remarks in Section 5. 

## 2. System Optimization 

**Figure 2:**
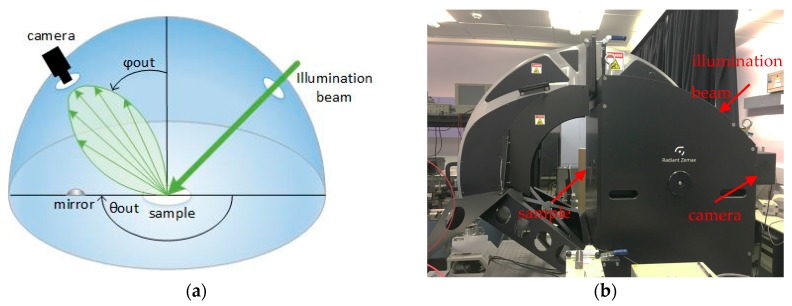
Imaging sphere (**a**) configuration where θ_out_ and φ_out_ shows the azimuth and elevation angles of reflected radiation; (**b**) Photograph.

In order to optimize the system in terms of best positioning of all the critical components (LEDs, LFIA, and array of photodiodes) and use the optimum number of LEDs, a ray trace simulation program (Zemax OpticStudio) [20] was used. In this program, all the components were modeled based on their actual size and their optical behavior. However, an LFIA has porous surface that will also be wet during the measurements and its optical behavior is not well known. The LFIA’s surface light behavior using an imaging sphere as shown in Figure 2 was characterized. 

The imaging sphere (from Radiant Vision Systems) measures the bi-directional scattering distribution function (BSDF) data for the precise modelling of a surface of a material [21]. It uses a light beam to illuminate the surface at various angles and then measures the scattered light from the surface to a camera. Due to the highly reflecting coating on the inside of the sphere and the convex mirror, the camera is able to measure the reflected radiation distribution over a full hemisphere (azimuth and elevation angle) [22]. A BSDF is generated in the form of tabular data which defines the scattering properties of the surface. For an accurate model of the LFIA using the imaging sphere, a test strip was used after Influenza A nucleoprotein in high concentration was applied. The illumination beam used had green wavelength because the gold nanoparticles present in the tested strip have diameter 20 nm and therefore highest light absorption in green wavelength. Using that strip, two BSDF models were generated; one for the white part of the strip and one for the part of the strip with the test line (the control line will have a similar response as the test line). 

**Figure 3:**
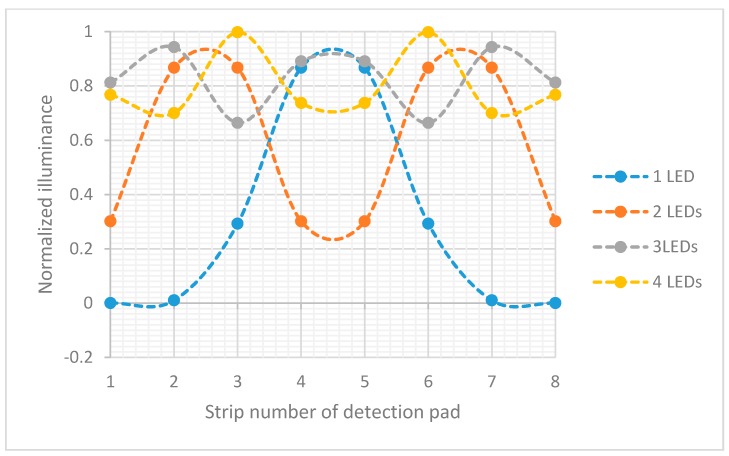
Normalized illuminance in each of eight 1 mm detection pad strips when the illumination is performed by varying the number of LEDs from one to four.

The optical characteristics of the LFIA surface, LEDs, and relevant dimensions were simulated using OpticStudio software (Zemax LLC, Washington, DC, USA). This optical design software was used for the simulation and optimization of the system based on the analysis of the optical behavior of all the components. First, the optimum number of LEDs needed to uniformly illuminate the detection pad of LFIA was defined. In order to do that, the 8 mm detection pad area (without any test or control lines) of LFIA was divided into eight distinct equal areas (1 mm each) and was placed above the LEDs (*dz* = 2 mm) and the array of photodiodes, in a similar configuration as shown in Figure 3. The total power per mm^2^ that strikes each area on the strip was measured and the maximum variation in lumens per mm^2^ per area was calculated. The same measurements were performed for varying number of LEDs (one to four). Figure 3 shows the normalized illuminance for each area in the detection pad. The LEDs had 525 nm wavelength and were equally spaced along the *y*-axis to cover the detection pad area. The smallest variation are about the same for three and four LEDs. To minimize the power used, three LEDs were chosen.

**Figure 4:**
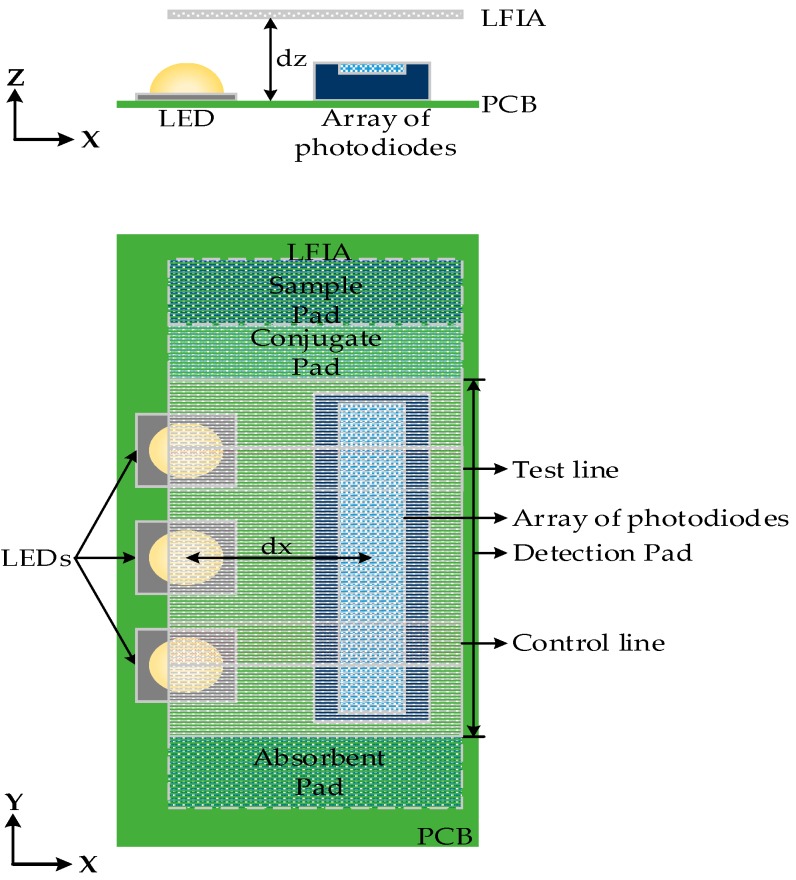
Simulation setup: plan and elevation (not to scale).

**Figure 5:**
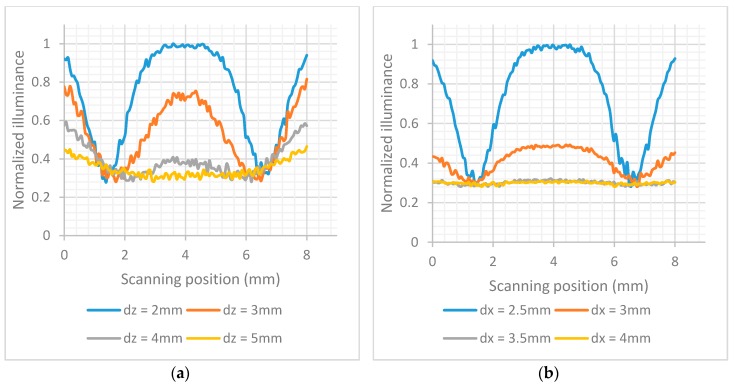
(**a**) Normalized illuminance across the array of photodiodes when the *dz* varies from 2 mm to 5 mm and three LEDs are used; (**b**) Normalized illuminance across the array of photodiodes when *dx* varies between 2.5 mm and 4 mm.

The detection pad of LFIA was designed to be placed above the array of photodiodes (see Figure 4). The construction was simulated in OpticStudio. In order to examine the optimum distance with respect to the array of photodiodes in *z*-axis a parametric analysis was performed for *dz* = 2 mm to 5 mm. The simulated LFIA had test and control lines with surface reflection properties based on the extracted BSDF data of the imaging sphere. The center of test line and the center of control line are 5 mm apart as in the practical test strips used. As shown in Figure 5a, the further away the LFIA is from the array of photodiodes, the more the light diffuses in more pixels and the less distinguishable are the lines in the strip. The different parts of the strip (test line, white part, control line) are more distinct when *dz* is 2 mm (this was the minimum practical distance). 

The illuminance striking the photodiodes was simulated for different values of *dx* when *dz* = 2 mm. The results are shown in Figure 5b. The most sensitive detection of the test and control lines is when *dx* = 2.5 mm. It should be noted that all the distances are measured from the center of LEDs to the center of array of photodiodes, and a distance less than 2.5 mm was not considered possible due to component sizes and PCB manufacturing constraints. 

**Figure 6:**
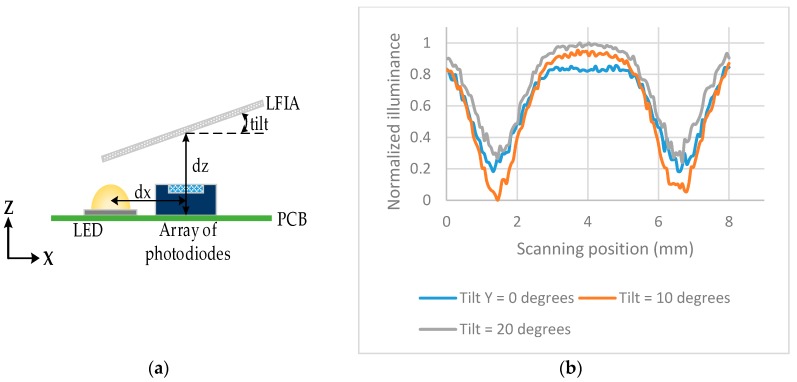
(**a**) Details of the tilt position, *dx* = 2.5 mm and *dz* = 2 mm; (**b**) normalized illuminance across the array of photodiodes when the strip is tilted in *y*-axis from 0° to 20°.

The illuminance across the array of photodiodes was also investigated when the LFIA is tilted in the *y*-axis by 0°, 10°, 20°. The tilt is performed from the center of the LFIA, therefore that point has a constant *dz* = 2 mm. The same setup as in Figure 4 was used but the LFIA was tilted as shown in Figure 6a. Figure 6b shows the largest difference (highest sensitivity) between the peaks and valleys of the illumination is for a tilt of 10°. 

## 3. Instrumentation

The prototype LFIA reader was realized based on the specifications from the optimization model discussed in Section 2. All the components that have been used are commercially available. The three LEDs (Kingbright) have a wavelength of 525 nm which is an approximate match to the absorption characteristics of 20 nm nanoparticles in the tested strips. The quasi monochromatic light is sufficiently narrow so that the absorption characteristics of the nanoparticles may be reduced by less than about 5%. Their dimensions are 1.6 mm × 0.8 mm × 0.95 mm each, which fit well to the requirements of the model. The maximum optical power of the LEDs was adjusted so that the light falling on the array of photodiodes does not saturate the pixels and provides a wide dynamic output range. The measurement method is based on the ratio of the signal in the test line to the signal in the control line, and to a first order the result is independent of LED optical power variations due to diode or power supply variation.

**Figure 7:**
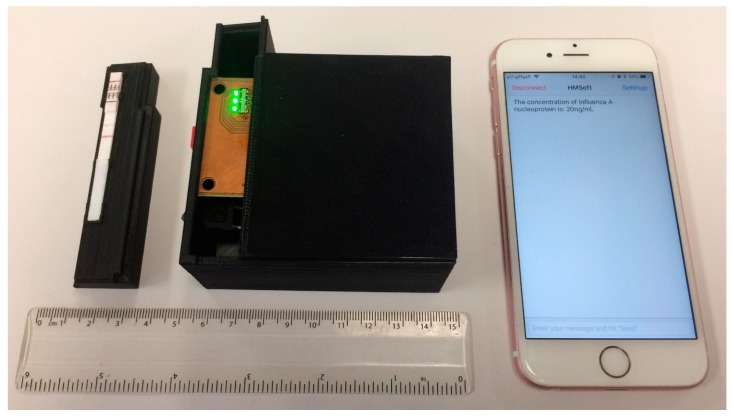
Photograph of the device. The lid is open, showing the carrier where the LFIA is placed fixed in the appropriate position.

For a light detector, a line of 128 photodiodes extending over 8 mm (AMS-TSL1401CL) was used. The chip processes and converts the photocurrents into output voltages from each pixel. The analog output was then digitized into the 10-bit analog-to-digital converter (ADC) of the microcontroller (ATmega). Further processing of the signal and the calculation of the concentration of analyte present in the sample was performed using the microcontroller. All the digital clocks needed for the correct operation of the system were also generated by the microcontroller. The final results were sent wirelessly using a low-power Bluetooth module to an open source app of a smart phone. All the electronic components were enclosed in a box specifically designed for this application using a 3D printer. A special carrier with small clips was designed for the LFIA so that the strip could be placed in the right position in the reader (above the array of photodiodes) with 10° tilt as simulated in the ray trace analysis program. Figure 7 shows a photograph of the developed device. The material of the box is black ABS with dimensions around 85 mm × 70 mm × 49 mm and it weighs 128 grams. With the appropriate power management and assuming each test takes 12 s, a standard PP9 9V can read and transmit about 5600 tests.

## 4. Measurements

**Figure 8:**
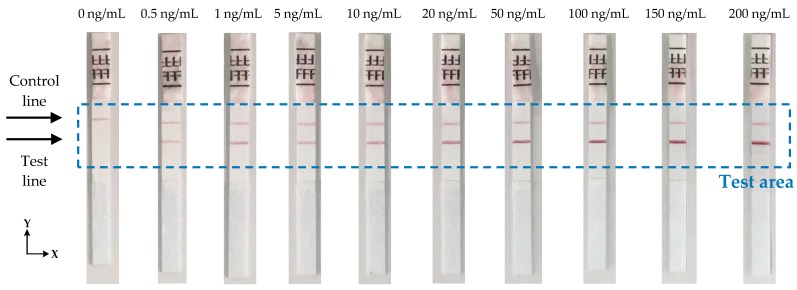
Photographs of the LFIAs in various concentrations that have been used for the testing of the system.

**Figure 9:**
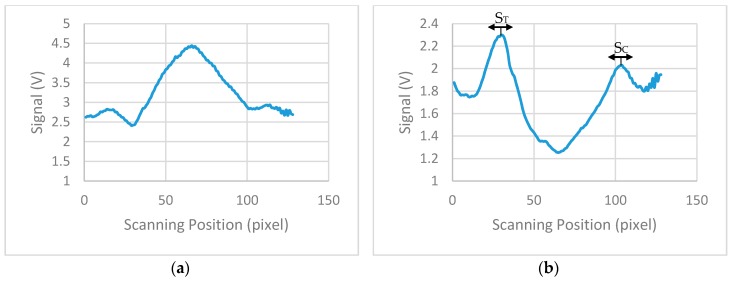
Lateral flow immunoassay with 20 ng/mL concentration of Influenza A nucleoprotein: (**a**) Voltage output of the 8 mm 128 photodiodes chip; (**b**) Voltage output signal after the implementation of background correction using a blank strip showing positions of S_T_ and S_C_.

The performance of the device has been experimentally evaluated using test strips from BD Directigen [23] in the concentration range of 0.5 ng/mL to 200 ng/mL of Influenza A nucleoprotein in saline buffer as shown in Figure 8. Each strip was measured 10 times to check the reproducibility of the reader and the average signal from each strip was calculated. Figure 9a shows the average calculated signal from a test strip with 20 ng/mL concentration. Background correction was performed by subtracting the signal of a blank strip (no lines, only saline buffer in the sample) to the average calculated signal. Figure 9b shows the calculated signal for the strip of 20 ng/mL concentration after the background correction. Due to implementation of this correction, the data reversed compared to measures data from the device and the maximum values represents the signal from the test and control lines. From the derived signals, the maximum value was detected for the first 20 to 50 pixels (124 pixels scan 8 mm) of each strip and then the average value of this maximum and the maxima of the ±4 adjacent pixels calculated. The calculated value corresponds to the signal of the test line (S_T_). The overall average value of 9 pixels was used because it is the number of pixels that approximately collect most of the reflected light from the test line. The same procedure was performed for the calculation of the signal of control line (S_C_), but the maximum was this time scanned between the corresponding pixels 90 to 120. The ratio S_T_/S_C_ was calculated for all the LFIAs shown in Figure 8 and is summarized in Table 1.

The effect of line positioning variation has been eliminated in the proposed design. The special carrier with small clips (Figure 7) ensures that the strip is always placed in the device in the right position. The automatic detection of the test and control lines eliminates any variations in the position of the lines caused by the fabrication process due to temperature, humidity, or air flow. Measurements show that the variation in positions of the lines is not more than 1.5 mm which is within the scanning range that the device is programmed to detect for each line (≈30 pixels = 1.875 mm).

**Figure 10:**
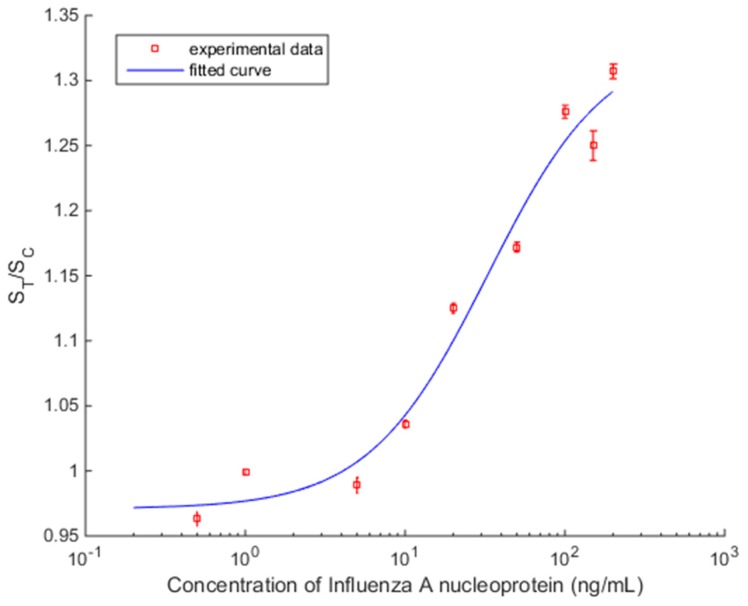
The fitted curve of S_T_/S_C_ for various concentrations of Influenza A nucleoprotein based on the 4PL model. The error bars indicate the ±2σ limits.

Figure 10 shows the plot of S_T_/S_C_ versus Influenza A concentration with spread ±2σ, which represents around 95% of the data distribution. It demonstrates consistent readings for repeated measurement for the same strips, but the presence of two concentrations which are outside the general trend (1 ng/mL and 150 ng/mL) suggest LFIA strip variation. Further statistical study of the performance of the LFIA strip is required to evaluate the accuracy of measurements. 

The concentration response curve of the analyte in the samples employed the commonly used four parameter logistic (4PL) model [19] shown in (1). It is used in many biological systems for analyzing the concentration of analyte [18].

(1) $$y=d+\frac{a-d}{1+{\left(\frac{x}{c}\right)}^{b}}$$

where *y* is S_T_/S_C;_ the measured signal, *x*, is the concentration of analyte; *a* is the response in minimum concentration; *d* is the response in maximum concentration; *c* is the concentration of analyte for the point halfway between *a* and *d*; and *b* the slope parameter. Based on the measured values of Table 1, MATLAB (MathWorks, Massachusetts, USA) was used to compute all the parameters of (1). The fitted curve is shown in Figure 10, with coefficient of determination *R*^2^ = 0.974 and the calculated parameters are *a* = 0.97, *b* = 1.16, *c* = 32.95, and *d* = 1.33.

The value of the cut-off signal has been calculated using a sample with no concentration of analyte of interest (0 ng/mL, only control line). The strip with this sample has been tested 10 times with the mean signal value being S_T_/S_C_ = 0.704 and the standard deviation σ = 0.0058. Therefore, the cut-off value that defines when the analyte of interest is not present in the sample is calculated to be S_T_/S_C_ + 3σ = 0.7214.

The reader device can detect samples with concentrations of Influenza A nucleoprotein as low as 0.5 ng/mL which is much less than the detection limit of the device in [8]. For the test of Influenza A nucleoprotein, the most important factor is the limit of detection rather than the resolution. This is because the early detection of Influenza is of more interest to the clinician than the exact concentration. Optimization determined that the lowest number of LEDs needed is three, limiting power consumption. Table 2 summarizes the features and performance of the device. The sole use of electronic components in the reader with no added optical components etc. can provide a very low cost device. 

## 5. Conclusions

A prototype readout device for LFIA has been developed. It has been tested using LFIAs with Influenza A nucleoprotein in the range of 0 ng/mL to 200 ng/mL concentrations. The BSDF model that characterizes the surface of LFIA has been experimentally measured using the imaging sphere. The model has been used in the ray trace simulations for the optimization of the system in order to increase its sensitivity using the minimum number of LEDs. An important characteristic of the reader proposed is that it automatically detects the line positions and therefore the results are not affected by variations of their width or positions on the strip. This resulted in consistent results with repeated measurements on the same LFIA strip. This offers the potential of quantitative measurements once the statistics of the LFIA paper strip are identified.

The concentration response curve was generated using the 4PL model. The device is able to detect Influenza A nucleoprotein in concentrations as low as 0.5 ng/mL. In the prototype, using a single PP9 battery, around 5600 measurements can be performed and its small robust build makes it an attractive reader for developing countries.

## Figures and Tables

**Table 1:** Concentrations of Influenza A nucleoprotein versus the measured signal S_T_/S_C_.

Concentration of Influenza A Nucleoprotein in (ng/mL)	S_T_/S_C_	Coefficient of Variation
0.5	0.963	0.28%
1	0.999	0.08%
5	0.989	0.33%
10	1.036	0.14%
20	1.125	0.16%
50	1.172	0.16%
100	1.276	0.19%
150	1.250	0.45%
200	1.307	0.21%

**Table 2:** Features and performance of the reader device.

Parameters	Value
Detection limit	0.5 ng/mL of Influenza A nucleoprotein
Wireless connectivity	Bluetooth 2.4 GHz
Prototype size	≈85 mm × 70 mm × 49 mm
Prototype weight	128 g
Measurements using a single PP9 battery	≈ 5600
